# Visual Impairment as a Function of Visual Acuity in Both Eyes and Its Impact on Patient Reported Preferences

**DOI:** 10.1371/journal.pone.0081042

**Published:** 2013-12-05

**Authors:** Robert P. Finger, Eva Fenwick, Christoph W. Hirneiss, Arthur Hsueh, Robyn H. Guymer, Ecosse L. Lamoureux, Jill E. Keeffe

**Affiliations:** 1 Center for Eye Research Australia, University of Melbourne, Royal Victorian Eye and Ear Hospital, Melbourne, Australia; 2 Department of Ophthalmology, University of Bonn, Bonn, Germany; 3 Singapore Eye Research Institute, National University of Singapore, Singapore, Singapore; 4 Ludwig-Maximilians University, Department of Ophthalmology, Munich, Germany; 5 Centre for Health Policy, Programs & Economics, School of Population Health, University of Melbourne, Melbourne, Australia; Zhongshan Ophthalmic Center, China

## Abstract

**Purpose:**

To assess the impact of VA loss on patient reported utilities taking both eyes into account compared to taking only the better or the worse eye into account.

**Methods:**

In this cross-sectional study 1085 patients and 254 controls rated preferences with the generic health-related (EQ-5D; n = 868) and vision-specific (Vision and Quality of Life Index (VisQoL); n = 837) multi-attribute utility instruments (MAUIs). Utilities were calculated for three levels of VA in the better and worse eyes, as well as for 6 different vision states based on combinations of the better and worse eye VA.

**Results:**

Using the VisQoL, utility scores decreased significantly with deteriorating vision in both the better and worse eyes when analysed separately. When stratified by the 6 vision states, VisQoL utilities decreased as VA declined in the worse eye despite stable VA in the better eye. Differences in VisQoL scores were statistically significant for cases where the better eye had no vision impairment and the worse seeing fellow eye had mild, moderate or severe vision impairment. In contrast, the EQ-5D failed to capture changes in better or worse eye VA, or any of the six vision states.

**Conclusions:**

Calculating utilities based only on better eye VA or using a generic MAUI is likely to underestimate the impact of vision impairment, particularly when the better eye has no or little VA loss and the worse eye is moderately to severely visually impaired. These findings have considerable implications for the assessment of overall visual impairment as well as economic evaluations within eye health.

## Introduction

Assessing the impact of visual impairment and blindness is met with the unique challenge that most people have two eyes with different levels of visual acuity (VA) which contribute to overall visual function. Therefore, functional impairment which affects both eyes differently is difficult to quantify overall. Economic evaluations frequently use utilities based on the better eye or differentiate between treatment of the worse and/or better eye,[1] assuming a differential impact on patients’ preferences (utilities) and quality of life (QoL), resulting in differing cost implications and cost-benefit ratios.[2] The most commonly used definition of blindness and visual impairment, published by the World Health Organization, is based on better eye VA, and the Global Burden of Disease Study estimates the global impact of visual impairment based on the better eye only.[3] Often, patients’ preferences are not directly elicited from the patients but inferred from better eye VA.[4] The better eye is assumed to predominantly determine daily visual functioning. However, this disregards the considerable loss of visual field, depth perception, as well as the anxiety caused by only having one seeing eye. Even unilateral vision loss has been shown to reduce independence considerably.[5] Similarly, use of the better eye only disregards the considerable impact of vision-restoring treatment in the worse eye, as achieved by for example cataract surgery or anti-VEGF treatment for neovascular age-related macular degeneration.[6] Past guidelines, for example, have recommended that treatment for disorders affecting both eyes are only made available for the better eye, as demonstrated by the 2007 draft “Guidance on the use of Lucentis® in neovascular age-related macular degeneration” published by the National Institute for Health and Clinical Excellence (NICE) in the UK.[7] To date, very little data on the differential impact of combined VA for better and worse eyes on utilities are available.[8] In order to better capture and understand patient preferences and other patient reported outcomes, a more detailed assessment of the association of vision in both eyes and reported preferences is warranted.

One way of capturing utilities is through multi-attribute utility instruments (MAUIs) in which values are indirectly elicited through patient ratings of their health status from a multi-featured classification system, which allows the comparison of utilities across different disease states. In Europe, the most commonly used MAUI is the European Quality of Life Questionnaire (EQ-5D) which is very well validated and widely available.[9]–[14] An alternative to the generic EQ-5D is the Vision and Quality of Life Index (VisQoL) which is a 6-item vision-specific MAUI developed and validated specifically for vision-impaired populations.[15] Time-trade off (TTO), a more direct way to elicit utilities by asking how much remaining life time a person is willing to trade in return for perfect health or perfect vision, has been found to be very sensitive in particular to small changes in vision-related utility.[6], [16] However, as it requires a face to face interview, and can pose a concept difficult to understand, we chose the easier to administer and comprehend MAUIs. The EQ-5D and the VisQoL were chosen to capture general and vision specific utility, respectively.

Based on the argument laid out above, we hypothesize that assessing vision and visual loss in both eyes allows for a more robust estimate of utility. Thus we assessed the impact of VA loss on patient reported utilities taking both eyes into account compared to taking only the better or the worse eye into account, using the EQ-5D and the VisQoL MAUIs.

## Methods

All patients were recruited from the outpatient clinic at the department of ophthalmology, University of Munich, Germany, and the Royal Victorian Eye and Ear Hospital, Australia, between 2009 and 2012. The only exclusion criteria applied was inability to partake in the interview, based on an inability to speak and/or read English or German or to comprehend the questionnaire. Fully sighted persons without any ocular pathology who were examined as part of a workplace screening intervention were included as controls, in order to reflect preferences by persons without any ocular disease or visual impairment, who did not seek medical care for any visual complaints, as an approximation to the general population. As anyone attending the respective hospital during recruitment periods was included, the patient sample is representative of persons seen at a tertiary eye hospital in Germany or Australia, respectively. Every participant underwent vision testing (see below), and a complete ophthalmic examination. Participants were given the MAUIs for self-completion. Institutional review board approval was obtained from the University of Munich and the Royal Victorian Eye and Ear Hospital. All patients gave signed informed consent for study participation and all studies adhered to the tenets of the Declaration of Helsinki.

### Creation of Vision States

Visual acuity was measured using best correction and either Early Treatment Diabetic Retinopathy Study (ETDRS) or logarithm of the minimum angle of resolution (LogMAR) retroilluminated charts. All VA data were transformed into the logMAR notation for subsequent analyses. Three categories of vision impairment (VI) were calculated for the better and worse eyes, individually (**Table 1**): (1) No VI (VA ≥6/12); (2) mild VI (<6/12–6/18); (3) moderate to severe VI (VA <6/18). In addition, with three categories of vision impairment in each eye, a combination of six different vision states were determined based on the three categories of VI in each eye (shaded areas in **Tables 2** **&** **3** **)**. For example, participants with one good eye (no VI) were grouped into either of the following three vision states based on the VA of the other eye: Vision state 1 with good VA in both eyes (VA ≥6/12), vision state 2 with good VA in one eye and mild VI (<6/12–6/18) in the second eye, or vision state 3 with good VA in one eye and moderate to severe VI in the second eye (VA <6/18). Patients with mild VI in their better eye were grouped in either vision state 4 (mild VI in the fellow eye) or vision state 5 (moderate – severe VI in the fellow eye). Patients with moderate – severe VI in both eyes were grouped into vision state 6.

**Table 1:** Characteristics of the sample.

		All n = 1339	EQ-5D n = 868	VisQoL n = 837
		n (%) or mean ± SD		
**Country**	Germany	796(60%)	387(45%)	606(72%)
	Australia	543(40%)	481(55%)	231(28%)
Age*		62.1±15.0	65.7±12.0	60.2±15.9
Gender*	Male	631(46%)	471(54%)	353(42%)
	Female	680(50%)	372(43%)	459(55%)
**Participant**	Patient	1085(80%)	868(100%)	583(70%)
	Control	254(20%)	0	254(30%)
Ocular condition* (patients only)	AMD	243(23%)	157(18%)	170(29%)
	DR/DME	730(67%)	620(71%)	308(53%)
	Other	109 (10%)	88(10%)	102(18%)
**Better eye VA (LogMAR)**		.24±.32	.22±.28	.23±.33
**Better eye VA categories**	6/12 or better no VI	893 (66%)	558(64%)	577(69%)
	<6/12–6/18 mild VI	209(15%)	154(18%)	102(12%)
	<6/18 moderate - severe VI	208(15%)	137(16%)	128(15%)

AMD = age-related macular degeneration; DR = diabetic retinopathy; DME = diabetic macular oedema; VA = visual acuity; VisQoL = Vision and Quality of Life Index; EQ-5D = Euro Quality of Life Questionnaire; LogMAR = logarithm of the minimum angle of resolution.*data incomplete.

**Table 2:** VisQoL utility scores by better or worse eye category and per vision state (by VA category).

				VA categories worse eye		
Better eye VA categories			Better eye only	6/12 or better	<6/12–6/18	<6/18
				No VI	Mild VI	Moderate – Severe VI
	6/12 or better	No VI	.92±.13* n = 577	**.95±.10 n = 371**	.90±.16* n = 111	.86±.17* n = 95
	<6/12–6/18	Mild VI	.84±.18* n = 102		**.85±.17 n = 32**	**.84±.19 n = 70**
	<6/18	Moderate –Severe VI	.71±.28*n = 128			.71±.28**n = 128**
**Worse eye only**				.95±.10*n = 371	.89±.17*n = 143	.79±.24*n = 293

VA = visual acuity, VisQoL = Vision and Quality of Life Index, *indicates significant difference (p≤0.001) between categories using post-hoc Bonferroni testing following ANCOVA. Bolded data correspond to the 6 vision states relating to combined categories of VI in the better and worse seeing eyes.

**Table 3:** EQ-5D (NZ VAS weighting) utility scores by better or worse eye category and per vision state (by VA category).

				VA categories worse eye		
Better eye VA categories			Better eye only	6/12 or better	<6/12–6/18	<6/18
				No VI	Mild VI	Moderate – Severe VI
	6/12 or better	No VI	.71±.23 n = 558	**.70±.22 n = 247**	**.71±.24 n = 170**	**.73±.23 n = 141**
	<6/12–6/18	Mild VI	.69±.23 n = 154		**.68±.23 n = 47**	**.70±.24 n = 107**
	<6/18	Moderate –Severe VI	.67±.23 n = 137			**.67±.23 n = 137**
**Worse eye only**				.70±.22 n = 247	.70±.24 n = 217	.70±.23 n = 385

VA = visual acuity, EQ-5D = Euro Quality of Life Questionnaire, VAS = visual analogue scale, NZ = New Zealand value set. Bolded data correspond to the 6 vision states relating to combined categories of VI in the better and worse seeing eyes.

### Patient-reported Preferences

#### Generic health-related patient-reported preferences – EQ-5D

The EQ-5D is a descriptive system that covers five dimensions of self-reported health: mobility, self-care, usual activity, pain/discomfort, and anxiety/depression. [9] Each dimension has three response categories: no problems, some problems and extreme problems. For example, a result of 11222 indicates no problems with mobility and self-care but some problems with the other three dimensions. The 243 health states defined by the EQ-5D responses were translated into EQ-5D index utilities using available values sets that have been derived from large population-based surveys.[17], [18] The scale of the utility index ranges between 0.0 and 1.0, where 0.0 represents death and 1.0 represents full health. States that are considered ‘worse than death’ are represented by negative utility values. Since no value set currently exists for the Australian population, or a mixed Australian and German sample we used the New Zealand value set [18] (VAS valuation method) as more Australian participants rated the EQ-5D.

#### Vision-related patient-reported preferences – the VisQoL

The VisQoL is a descriptive system that covers six dimensions of self-reported vision-related quality of life (VRQoL): physical well-being, independence, social well-being, self-actualisation, and planning and organisation. [15], [19] Each question is preceded by “Does my vision…” and each dimension has between five and six response categories, ranging from, for example, ‘no effect’ to ‘unable to do’. Two dimensions also have a ‘non-applicable’ option. The health states defined by the VisQoL responses were translated into VisQoL utilities using an available value set derived from surveys using the TTO method.[15] The value set was generated as part of the instrument validation, using a range of health states and a visually impaired as well as a visually unimpaired sample.[19] Item utilities were combined using a multiplicative model and the scale of the utility index ranges from 0–1, where 0.0 represents the worst imaginable vision-related health state, i.e. blindness, and 1.0 represents the best imaginable vision-related health state, i.e. perfect vision. Health states rated worse than blindness are represented by negative utility values.

### Statistical Analyses

The SPSS statistical software (Version 19.0, SPSS Science, Chicago, IL) was used to analyze the data. Participants with missing visual acuity data were excluded from all analyses. Other missing data were not inferred, but patients excluded from the respective analyses. Descriptive statistical analyses were performed to characterize the participants and their utilities. The correlation between utility measures and participants’ characteristics were explored using Spearman’s rank correlation. Utility measures were not compared directly. For each utility instrument (VisQoL and EQ-5D), utilities per vision state and across better and worse eye VI categories were compared using using post-hoc Bonferroni testing following analysis of covariance, controlling for age and gender (ANCOVA). All tests were considered to be statistically significant at a level of p<0.05 and corrections were made for multiple testing.

## Results

The overall sample consisted of 1085 patients and 254 fully sighted controls without any ocular pathology, of which 543 were interviewed in Australia and 796 in Germany. Mean ± standard deviation (SD) age was 62±15 years, with equal gender proportions (50% female, **Table 1**). The mean (±SD) VA in the better eye was 0.24±0.32 (LogMAR). A total of 837 respondents rated the VisQoL, and 868 rated the EQ-5D. Of these, 384 rated both instruments. Overall, the majority of patients had diabetic eye diseases (diabetic retinopathy and/or diabetic macular edema; n = 730, 67%), followed by age-related macular degeneration (n = 243, 23%), and other ocular diseases such as cataract and glaucoma (n = 109, 10%). The German and the Australian patient samples did not differ except for the proportion of persons with diabetic eye disease which was larger in the Australian sample (p<0.05). Controls did not differ in gender proportions from patients, but were younger (46±9 versus 66±13 years; p<0.001) and had a better VA (0.03±0.09 versus 0.28±0.33; p<0.001).

All utility scores correlated with age (all p<0.03), but only VisQoL utilities were correlated with VA for better and worse eyes (r = −0.451 and r = −0.481, respectively, both p<0.001).

**Figure 1:**
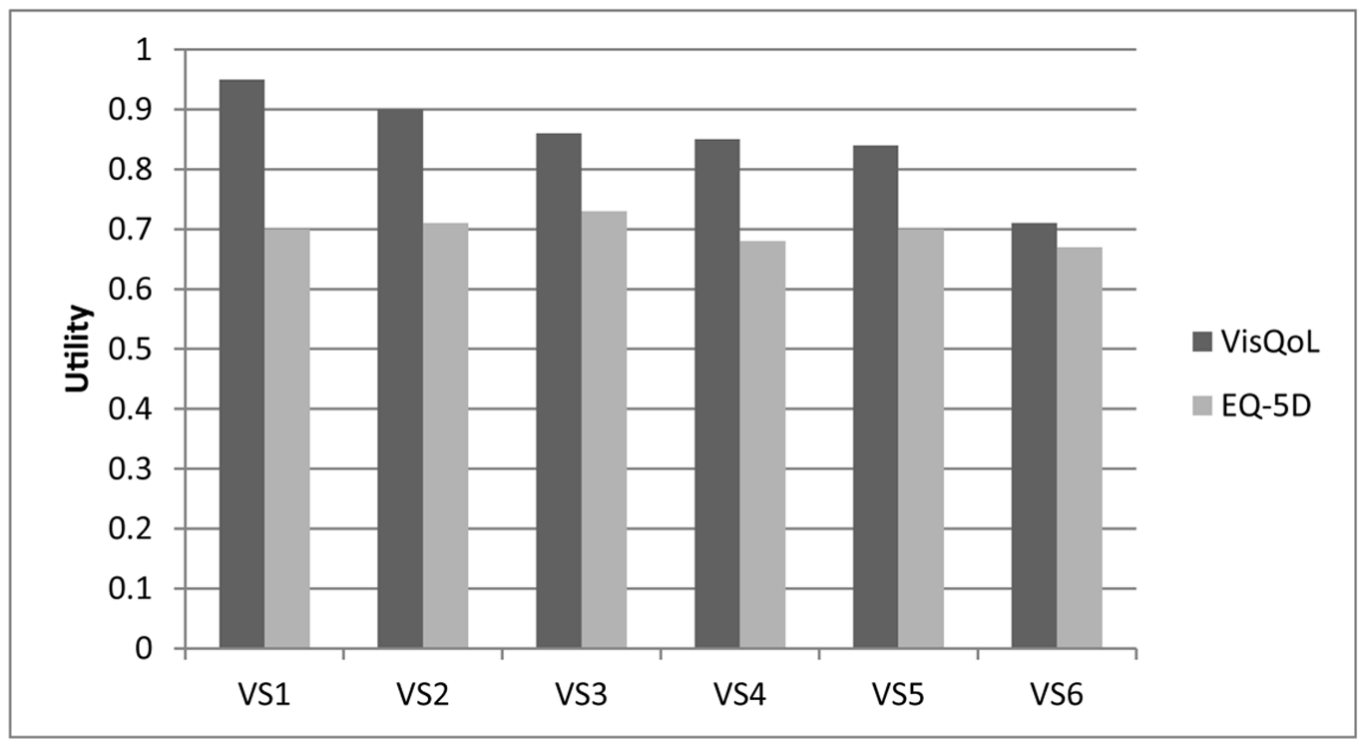
Utility across all six vision states (VS), for the VisQoL and the EQ-5D utility instruments, demonstrating a reduction of visual acuity with worsening vision states for the VisQoL but not the EQ-5D.

Using the VisQoL, utility scores decreased significantly with deteriorating vision in both the better and worse eyes, separately (all p<0.001, **Figure 1**, **Table 2**). Considering the different vision states based on both eye VA, reported VisQoL utilities decreased with deteriorating vision in the worse eye despite no change in the better eye (rows in shaded area in **Table 2**). This was statistically significant for mild (<6/12–6/18, p≤0.001) or moderate to severe (<6/18, p≤0.001) VI in the worse seeing fellow eye despite stable and very good vision in the better seeing eye (no VI >6/18, **Figure 1**, **Table 2**). In contrast, EQ-5D utilities did not differ according to better eye VA, worse eye VA or by vision states considering both eyes (**Figure 1**, **Table 3**).

## Discussion

Using the VisQoL, the use of better eye VA only, or VA of one eye only, may be suboptimal when determining utilities, particularly in cases with good better eye function and moderate to severe visual impairment in the fellow eye. Our data show that determination of vision-related utility values should be guided by VA in both eyes with specific attention given to the worse eye in cases with good seeing better eyes. This finding has considerable implications for defining visual impairment, burden of disease and economic evaluations within eye health as well as treatment decisions, as the conventional maxim “still got one good eye”, referring to basing treatment or other decisions on better eye VA, is unlikely to reflect patients’ preferences. We found that the EQ-5D generic MAUI was completely unresponsive to different VA levels in a large sample of patients as has been suggested by work from this group previously.[20] Conversely, the VisQoL - a vision-related MAUI – was better able to record utilities associated with vision impairment.

Contrary to the assumption that the better eye solely or mostly determines vision-related activity limitation, quality of life and utilities, treatment of the better *and* worse eye confers a patient reported benefit. In a previous study we demonstrated that improvements in utilities did not depend on whether the better or worse eye was treated in patients with neovascular AMD.[8] In large, pivotal phase III studies of treatment for neovascular AMD, the observed increase in visual functioning as measured with a vision-specific patient reported outcome measure, the National Eye institute Visual Functioning Questionnaire 25 items (NEI-VFQ-25), has been similar regardless of whether the better or worse eye was treated.[21] Similarly, surgical interventions for macular holes have been associated with an increase in VRQoL without a corresponding change in VA in the treated eye which, in most cases, remained the worse eye even after a successful intervention.[22] The findings from the current study of an additional impact of worse eye VA on reported utilities combined with the above evidence that improvements in utilities and VRQoL are often irrespective of better or worse eye treatment, suggest that resource allocation and treatment decisions should not be based on better eye VA only. Nor should treatments be made available for the better eye only in bilaterally affected cases. On the contrary, evidence strongly suggests that patients should have access to treatment and care the moment visual function in either eye is affected. Studies based on the better eye VA only are likely to under-estimate the impact of visual impairment and its overall burden. For example, the burden of visual impairment estimated by the Global Burden of Disease study [3] is likely to be higher as a large proportion of patients with no VI in their better eye but even only mild VI in their fellow worse seeing eye will be impacted and should be considered to contribute to the overall burden, as shown in our study. However, these cases are disregarded in the currently employed methodology.

Utility values used for economic evaluation in ophthalmology are often deducted using approximations from clinical data (e.g. VA of the better eye) rather than from directly collected utilities.[10], [13], [23], [24] Utilities directly derived from patients, and based on VA in both eyes, are very likely to differ from utility values inferred from VA of the better eye only. Collecting utilities directly from patients is more resource intensive than inferring them from VA, but likely to yield more differentiated and authentic health or vision states as well as corresponding utilities. One possible compromise could be to use a table similar to this study (e.g. **Table 2**), which reflects the VA of both eyes in combination. However, further research in another sample with a greater differentiation of ocular conditions as well as larger numbers is required to validate this methodology. This would allow to stratify VA into finer categories as well. Given these issues, it remains a challenge to find the optimal method of accurately assessing utilities related to vision impairment and treatment in ophthalmology which is specific yet comparable across different diseases and impairments.

Strengths of our study include the use of a standard generic MAUI and a vision-specific MAUI in a very large sample of more than 1000 patients recruited at two centres who underwent a complete ophthalmic examination and standardized vision assessment. The main limitation of our study is the small sample size of some of the six combined vision states, particularly those comprising poor VA in both eyes, which may have diminished our ability to reveal significant associations. As this was an exploratory study, we did not conduct formal sample size calculations to inform recruitment. Collating data collected in Australia and Germany may have limitations due to cross-cultural differences. Distance VA may not be the most appropriate clinical measure to represent functional vision, with a number of studies suggesting that contrast sensitivity may be more highly associated with visual functioning and utilities.[23], [25], [26] However, as best corrected VA is the most widely used and standardized measure in daily clinical routine this ultimately enhances the generalisability of our findings. Similarly, the somewhat crude categorization into three different levels of vision impairment may lead to a loss of information. Future studies should aim to recruit more participants which would then allow for a finer categorization of vision.

In conclusion, calculating utilities based only on better eye VA is likely to underestimate the impact of vision impairment, in particular when the better eye functions well and the other (worse) eye is moderately or severely visually impaired. These findings have considerable implications for defining visual impairment, for economic evaluations within eye health as well as for treatment decisions, as the conventional maxim “still got one good eye” is likely to not reflect patients’ preferences and underestimate the impact of poor vision in either eye.

## References

[1] Hurley SF, Matthews JP, Guymer RH (2008) Cost-effectiveness of ranibizumab for neovascular age-related macular degeneration. Cost Eff Resour Alloc 6: 12. 18573218 10.1186/1478-7547-6-12 PMC2443361

[2] Mitchell P, Annemans L, White R, Gallagher M, Thomas S (2011) Cost effectiveness of treatments for wet age-related macular degeneration. Pharmacoeconomics 29: 107–131. 21244102 10.2165/11585520-000000000-00000

[3] Bourne R, Price H, Stevens G (2012) Global burden of visual impairment and blindness. Archives of ophthalmology 130: 645–647. 22652851 10.1001/archophthalmol.2012.1032

[4] Sharma S, Brown GC, Brown MM, Shah GK, Snow K, et al (2000) Converting visual acuity to utilities. Canadian journal of ophthalmology Journal canadien d’ophtalmologie 35: 267–272. 10.1016/s0008-4182(00)80077-0 10959467

[5] Vu HT, Keeffe JE, McCarty CA, Taylor HR (2005) Impact of unilateral and bilateral vision loss on quality of life. Br J Ophthalmol 89: 360–363. 15722319 10.1136/bjo.2004.047498 PMC1772562

[6] Finger RP, Hoffmann AE, Fenwick EK, Wolf A, Kampik A, et al (2012) Patients’ preferences in treatment for neovascular age-related macular degeneration in clinical routine. The British journal of ophthalmology 96: 997–1002. 22535331 10.1136/bjophthalmol-2011-301201

[7] National Institute for Health and Clinical Excellence (NICE) (2007) NICE issues draft guidance on drugs for the treatment of age related macular degeneration. National Institute for Health and Clinical Excellence (NICE).

[8] Finger R, Hoffmann AE, Fenwick EK, Wolf A, Kampik A, et al.. (2012) Patients’ preferences in treatment for neovascular age-related macular degeneration in clinical routine. The British journal of ophthalmology. 10.1136/bjophthalmol-2011-301201 22535331

[9] Williams A (1990) EuroQol - a new facility for the measurement of health-related quality of life. Health Policy 16: 199–208. 10109801 10.1016/0168-8510(90)90421-9

[10] Brown GC (1999) Vision and quality-of-life. Trans Am Ophthalmol Soc 97: 473–511. 10703139 PMC1298275

[11] Brown MM, Brown GC, Sharma S, Garrett S (1999) Evidence-based medicine, utilities, and quality of life. Curr Opin Ophthalmol 10: 221–226. 10537783 10.1097/00055735-199906000-00012

[12] Brown MM, Brown GC, Sharma S, Busbee B, Brown H (2001) Quality of life associated with unilateral and bilateral good vision. Ophthalmology 108: 643–647; discussion 647–648. 10.1016/s0161-6420(00)00635-7 11297474

[13] Brown GC, Brown MM, Sharma S, Beauchamp G, Hollands H (2001) The reproducibility of ophthalmic utility values. Trans Am Ophthalmol Soc 99: 199–203; discussion 203–194. PMC1359010 11797307

[14] Hirneiss C, Rombold F, Kampik A, Neubauer AS (2006) [Visual quality of life after vitreoretinal surgery for epiretinal membranes]. Ophthalmologe 103: 109–113. 16078065 10.1007/s00347-005-1252-0

[15] Peacock S, Misajon R, Iezzi A, Richardson J, Hawthorne G, et al (2008) Vision and quality of life: development of methods for the VisQoL vision-related utility instrument. Ophthalmic Epidemiol 15: 218–223. 18780254 10.1080/09286580801979417 PMC2562021

[16] Hiratsuka Y, Yamada M, Murakami A, Okada AA, Yamashita H, et al (2011) Cost-effectiveness of cataract surgery in Japan. Jpn J Ophthalmol 55: 333–342. 21695383 10.1007/s10384-011-0041-3

[17] Dolan P (1997) Modeling valuations for EuroQol health states. Med Care 35: 1095–1108. 9366889 10.1097/00005650-199711000-00002

[18] Devlin N, Hansen P, Kind P, Williams A (2003) Logical inconsistencies in survey respondents’ health state valuations - a methodological challenge for estimating social tariffs. Health Economics 12: 529–544. 12825206 10.1002/hec.741

[19] Misajon R, Hawthorne G, Richardson J, Barton J, Peacock S, et al (2005) Vision and quality of life: the development of a utility measure. Investigative ophthalmology & visual science 46: 4007–4015. 16249474 10.1167/iovs.04-1389

[20] Fenwick EK, Xie J, Ratcliffe J, Pesudovs K, Finger RP, et al (2012) The impact of diabetic retinopathy and diabetic macular edema on health-related quality of life in type 1 and type 2 diabetes. Investigative ophthalmology & visual science 53: 677–684. 22205611 10.1167/iovs.11-8992

[21] Bressler NM, Chang TS, Suner IJ, Fine JT, Dolan CM, et al.. (2010) Vision-related function after ranibizumab treatment by better- or worse-seeing eye: clinical trial results from MARINA and ANCHOR. Ophthalmology 117: 747–756 e744. 10.1016/j.ophtha.2009.09.002 20189654

[22] Hirneiss C, Neubauer AS, Gass CA, Reiniger IW, Priglinger SG, et al (2007) Visual quality of life after macular hole surgery: outcome and predictive factors. The British journal of ophthalmology 91: 481–484. 17077117 10.1136/bjo.2006.102376 PMC1994732

[23] Bansback N, Czoski-Murray C, Carlton J, Lewis G, Hughes L, et al (2007) Determinants of health related quality of life and health state utility in patients with age related macular degeneration: the association of contrast sensitivity and visual acuity. Qual Life Res 16: 533–543. 17119846 10.1007/s11136-006-9126-8

[24] Bansback N, Davis S, Brazier J (2007) Using contrast sensitivity to estimate the cost-effectiveness of verteporfin in patients with predominantly classic age-related macular degeneration. Eye (Lond) 21: 1455–1463. 17086167 10.1038/sj.eye.6702636

[25] Holton H, Christiansen AB, Albeck MJ, Johnsen CR (2009) The impact of light source on discrimination ability in subjects with age-related macular degeneration. Acta Ophthalmol. 10.1111/j.1755-3768.2009.01809.x 20015100

[26] Espallargues M, Czoski-Murray CJ, Bansback NJ, Carlton J, Lewis GM, et al (2005) The impact of age-related macular degeneration on health status utility values. Invest Ophthalmol Vis Sci 46: 4016–4023. 16249475 10.1167/iovs.05-0072

